# Impact of regional accessibility on urban residents’ income in China: A bidirectional-fixed panel model

**DOI:** 10.1371/journal.pone.0289866

**Published:** 2023-10-30

**Authors:** Yahong Liu, Daisheng Tang, Fengyu Wang, Naila Bano

**Affiliations:** 1 Business School, Xuzhou University of Technology, Xuzhou, China; 2 School of Economics and Management, Beijing Jiaotong University, Beijing, China; Public Library of Science, UNITED KINGDOM

## Abstract

The income gap between regions and its expansion are the main manifestations of the imbalanced and inadequate economic development in China. High-speed railway (HSR) construction is regarded as an important method to drive domestic demand, drive the pulse of the economy, and promote the coordinated development of regions. Based on the opening of HSR and the acceleration of ordinary railways, we used the weighted average travel time model and accessibility coefficient to estimate the changes on accessibility in 286 cities at prefecture-level and above from 2000 to 2018. Then, the influence mechanisms of improving regional accessibility on urban residents’ income were estimated by using the bidirectional-fixed effects panel model and the recursive model respectively. We found that: (1) The accessibility of urban areas has been greatly improved due to the opening of HSR and the acceleration of ordinary railway, among which the improvement of HSR cities is greater. (2) The improvement of regional accessibility significantly promoted the income growth of urban residents, and the increase of the regional accessibility coefficient by 1 unit led to an average increase of 2140 yuan in the per capita disposable income of urban residents. (3) There is regional heterogeneity in the impact of improving regional accessibility on urban residents’ income, and it has a significant effect on the eastern and northeastern regions. It has a greater positive effect on improving the income of residents in central cities compared with peripheral cities. (4) Regional accessibility can promote urban income growth through regional employment and fixed asset investment. In the future, the transportation network should be further improved to facilitate the regional economic cycle, strengthen the coordination and complementarity of regional economies, and promote regional economic integration so as to promote the improvement of resident income level and the common prosperity of the people.

## Introduction

The income gap between regions and its widening phenomenon is the main form of income gap in China and also one of the main manifestations of the imbalance and inadequacy of the current economic development in China [1]. The report to the 19th National Congress of the Communist Party of China (CPC) pointed out that the principal contradiction facing Chinese society has been that between unbalanced and inadequate development and the people’s ever-growing needs for a better life. Since the reform and opening up (1978), the first rich have driven the second rich, and the development policy of achieving common prosperity has promoted the rapid development of China’s economy. By the end of 2018, China’s GDP exceeded RMB 90 trillion yuan, and its per capita income reached 28,200 yuan.

**Fig 1:**
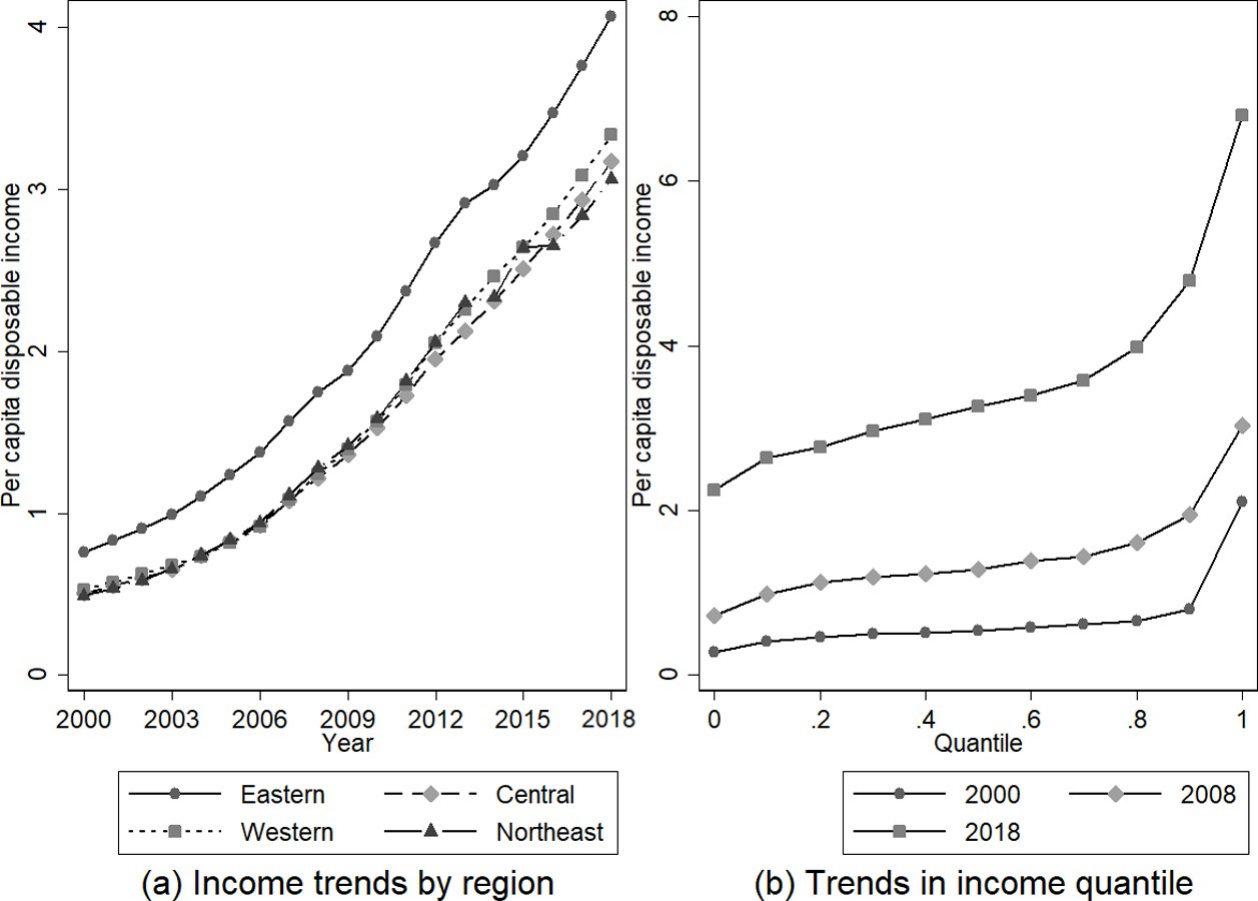
Variation trend of per capita disposable income of urban residents (ten thousand yuan) in 286 cities at prefecture level and above in China, 2000–2018. Note: ① "Cities at prefecture-level and above" are the second level of administrative divisions in China. 286 cities at prefecture-level and above were sorted out from The Statistical Yearbook of Chinese Cities over the years due to the changes in administrative divisions over the years and the serious lack in samples of some autonomous prefectures and cities. ② The per capita disposable income of residents is calculated as the aggregate of the final consumption expenditure and savings of residents. This encompasses the discretionary income available to residents, which comprises both monetary and non-monetary forms of income. According to the source of income, disposable income includes wage income, operating net income, property net income and transfer net income. The income of urban residents used in this paper refers to the per capita disposable income of urban permanent residents. The data of the per capita disposable income of urban residents from 2000 to 2018 was obtained from The Statistical Yearbook of Chinese Cities from 2001 to 2019. ③ Eastern China includes: Beijing, Tianjin, Hebei, Shanghai, Jiangsu, Zhejiang, Fujian, Shandong, Guangdong, and Hainan. Central China includes Shanxi, Anhui, Jiangxi, Henan, Hubei and Hunan. Western China includes: Inner Mongolia, Guangxi, Chongqing, Sichuan, Guizhou, Yunnan, Tibet, Shaanxi, Gansu, Qinghai, Ningxia, Xinjiang. Northeastern China includes: Liaoning, Jilin and Heilongjiang.

However, there is also an imbalance in economic development, industrial structure, culture, and education among regions, and there is a large gap in the income level of residents among cities across the country. From 2000 to 2018, the per capita disposable income of residents in eastern China was much higher than that of other regions (see Fig 1(A)). For example, in 2000, 2008, and 2018, the percentile curve of urban income became steeper, that is; the income difference between higher income cities and lower income cities increased over the years among 286 cities at the prefecture level and above (see Fig 1(B)). In 2000, residents’ income in the lowest city was 2,830 yuan, while that in the highest city was 20,990 yuan. In 2018, residents’ income in the lowest city was 22,470 yuan, while that in the highest city was 68,030 yuan. From the perspective of the spatial distribution of residents’ income, the income of residents in some central cities or cities with a higher administrative grade and a better economic foundation was at a higher level, especially in eastern cities with higher income levels. The income gap between cities is gradually widening, and there are fewer cities with higher income levels.

Transportation is essential to national development and the foundation of a strong country, and the construction of transportation infrastructure has become an important method to develop the space economy [2]. High-speed railway (HSR) generally refers to the railway system whose operation rate reaches above 200km/h through the transformation of the original line (linearization, gauge standardization) or a specially designed new railway system whose operation rate reaches above 250km/h (International Union of Railways, UIC). In this paper, the speed of transportation above 200km/h is defined as HSR. Since the 21st century, China has been using HSR construction as an important way to drive domestic demand, drive the economic pulse, and promote coordinated regional development [3]. From the "four vertical and four horizontal" and "eight vertical and eight horizontal" railway transportation networks to the "transportation power" and "promoting common prosperity of the people" (see Table 1), the role of transportation infrastructure construction in national economic development is self-evident. By the end of 2018, 204 of 286 cities at prefecture-level and above had opened HSR since the Beijing-Tianjin-Hebei intercity high-speed train was put into operation in 2008. China’s total railway mileage reached 131,700 km, with 29,000 km of HSR mileage, accounting for two-thirds of the world’s total. China has become the country with the longest HSR, the highest transportation density, and the most complex network operation in the world [4]. From 2008 to 2018, the number of HSR passengers increased from 7.34 million to 2054.3 million, and the proportion of HSR passengers in the total passenger traffic increased from 0.03 percent to 11.45 percent, indicating a rapid increase in the number of HSR passengers. In addition, the third (October 21, 2000), fourth (November 21, 2001), fifth (April 18, 2004) and sixth (200 km/h, April 18, 2007) speed increases were made for ordinary railways from 2000 to 2018. The formation of a relatively complete urban agglomeration with a comprehensive transportation network affects the speed and quality of regional economic development.

**Table 1:** Policy summary of China’s transportation power.

Date of Publication	Document	Major Topics
January 19, 2022	The 14th Five-year Plan for the development of modern comprehensive Transportation system	Transportation is a fundamental, pioneering, and strategic industry in the national economy. It is an important service industry and an important component of the modern economic system. It is an important support for building a new development pattern and a solid guarantee for serving the people’s better lives and promoting common prosperity.
March 11, 2021	Outline of the 14th Five-Year Plan for National Economic and Social Development and the Vision For 2035	We will ensure that personal income increases basically in step with economic growth and that labor remuneration increases in step with labor productivity increases. We will continue to raise the income of low-income groups, expand the middle-income group, and work more vigorously to promote common prosperity.
March 5, 2021	Government Work Report of The State Council in 2021	Optimize regional economic layout and promote coordinated regional development. Continue to improve people’s wellbeing and promote common prosperity. We will focus on increasing the income of low-income groups and expanding the middle-income group, and we will ensure that per capita disposable income increases basically in step with GDP growth.
February 24, 2021	Outline of national Comprehensive Three-dimensional Transportation Network Planning	By 2035, a modern and high-quality national comprehensive three-dimensional transportation network will be basically built, that is convenient, smooth, economical, efficient, green, intensive, intelligent, safe, and reliable. It will be interconnected with the rest of the world, accessible to major cities in China, and have effective coverage of county-level nodes. Transportation fully meets people’s growing needs for a better life.
September 19, 2019	Outline of Building a Transport Power	We will strengthen areas of weakness in the western region, improve the quality and upgrading of the northeast, promote the development of major transportation corridors and hubs in the central region, and accelerate the optimization and upgrading of the eastern region, so as to create a new pattern of coordinated regional transport development.
July 13, 2016	Medium and Long Term Railway Network Planning (2016–2030)	A high-speed railway network will be formed, with eight vertical and eight horizontal main passageways as the backbone and regional connection lines connected and supplemented by inter-city railways, so that provincial capitals will be connected by high-speed railways and inter-regional connections will be efficient and convenient.
October 08, 2008	Medium and Long Term Railway Network Planning (2008 Adjustment)	China’s high-speed railway development focuses on the "four vertical and four horizontal" special passenger lines with passenger cars running at speeds of more than 200 kilometers per hour, accelerating the construction of the main framework of the rapid passenger transport network.

Note: The data are from documents issued by the CPC Central Committee, The State Council, the Development and Reform Commission and the Ministry of Railways of the People’s Republic of China.

A modern railway network, with HSR as its backbone, has the potential to offer substantial transportation capacity while simultaneously promoting more balanced and adequate regional development. The reduction of transportation costs, the shortening of people’s travel time, the improvement of regional geographical location, and promotion the flow and rational allocation of resources such as human flow, logistics, and capital flow along the belt and road are expected to have a profound impact on regional factor aggregation and economic development [5].

Differences in transport infrastructure investment are also important factors in the widening of the inter-provincial economic growth gap in China [2, 6], the rapid transit system represented by HSR constantly reconstructs the trajectory of labor mobility and the spatial distribution pattern of regional economies. So, can the development of transportation infrastructure represented by the opening of HSR become an effective policy tool for China to adjust the income of residents between regions and narrow the income gap? The current stage of high-quality economic development in China is characterized by pressing needs such as promoting common prosperity, addressing the problem of unbalanced and inadequate development, narrowing the disparity between regional and urban development as well as income distribution, improving the quality and efficiency of development, and improving the standard of living of residents.

It is of great theoretical and practical significance to analyze the impact of the improvement of regional accessibility based on the construction of transportation infrastructure on residents’ income levels in order to narrow the income gap between regions and promote residents’ income and common prosperity. The most direct impact of transportation development on cities and regions comes from changes in regional accessibility [7]. Regional accessibility refers to the convenience of using a specific transportation system to get from a certain location to a specified location. It is the opportunity potential of interaction between nodes in the transportation network and can reflect the convenience of economic activities from a given location to the activity site by using a specific transportation system [8].

**Fig 2:**
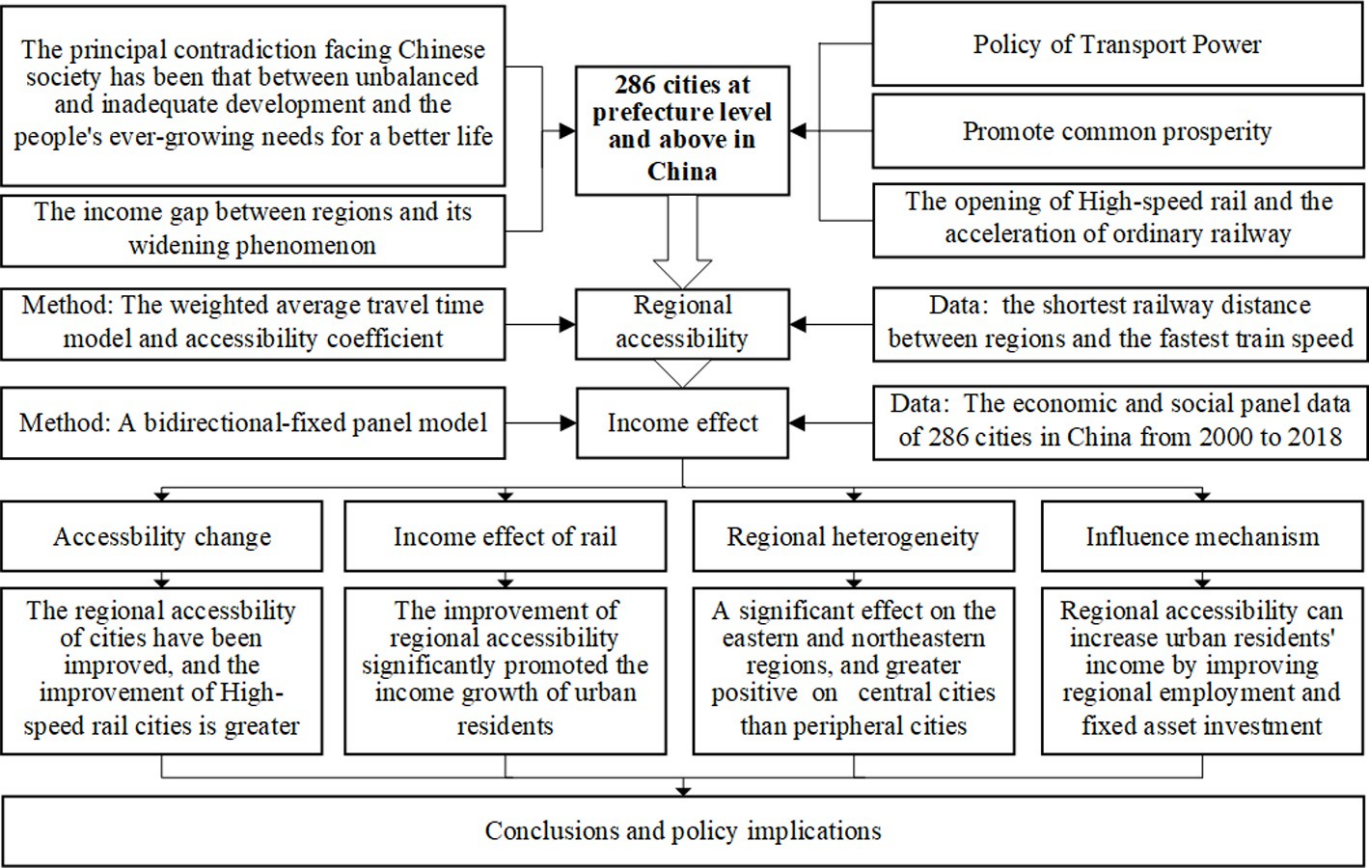
Frame diagram of this study.

This study aimed to assess the influence of enhanced regional accessibility on the income of urban residents. The analysis was conducted by examining the dynamic alterations in regional accessibility that resulted from the introduction of High-Speed Rail (HSR) and the acceleration of ordinary railway in 286 cities at the prefecture level and above in China between 2000 and 2018. The findings of this research could offer valuable insights and policy recommendations for the income effect theory and practice, regional economic integration, and the promotion of common prosperity through transportation infrastructure development in comparable countries worldwide. Fig 2 illustrates the framework utilized in this study.

## Literature review

The construction and operation of HSR can open up multiple axes of economic development, form a crisscrossing, efficient, and convenient HSR transportation network system, and change the regional economic development pattern from "point-axis mode" to "network mode", thus affecting the economic development of the whole region [9]. Accessibility is an important method to measure the structure and spatial distribution of transportation networks, and it can be used to investigate their social and economic value in terms of time, space, economy, and population flow.

Scholars studied the impact of HSR on accessibility across various spatial scales including transnational [10], national [11, 12], district (provincial) domain and circuit [13], urban interiors and sites [14, 15], as well as macro and micro level of analysis [16]. Some scholars further studied the impact of HSR on urban network structure and spatial interaction intensity based on the ArcGIS network analysis tool [17]. Accessibility measures based on the distance method mainly include weighted average travel time (WATT) [10, 18, 19], Location Advantage potential Model (PA) [20] and Daily accessibility (DA) [21]. The most significant feature of HSR is speed, and its running time is considered particularly important [11]. Different accessibility calculation models have different emphases because urban accessibility is influenced by geographical location, traffic conditions, economic development level, and city size. The potential model assumes the city as a point factor and pays attention to economic flow but neglects the discussion on economic quality and surface phenomenon [22]. Daily accessibility focuses on the population or economic scale within a certain time limit, focusing on economic benefits while weakening location and transportation costs [23]. The weighted average travel time method, based on economic weight, comprehensively considers the travel cost and location attractiveness. It can weaken the spatial barriers of geographical location, strengthen the connection between node economic scale and transportation cost, measure urban accessibility more accurately, and combine with the accessibility coefficient to explain the evolution of the spatial pattern of accessibility [24].

The development of transportation infrastructure improves the regional accessibility of cities along the routes, enhances the breadth and depth of inter-city or inter-regional economic connections, and leads to profound changes in regional spatial form, function, and development mode [25, 26]. Scholars have investigated the impact of transportation infrastructure on residents’ income by including the stock of transportation infrastructure into the income distribution model [27], taking the opening of HSR as a Difference-in-Difference model [28] and Panel data model [29]. Its influence path mainly includes the following four aspects: Firstly, the improvement of regional accessibility brought by the opening of HSR promotes the convenience of urban transportation, produces boundary breakthrough and location enhancement effects, and expands the spatial boundary of the circulation of production factors such as capital, resources, and intermediate products [30]. The saving of time and cost can create more additional value, optimize the allocation of production factors in a larger geographical space, improve the efficiency of economic operation, and promote the increase of residents’ income [31]. Secondly, the convenience of transportation promotes a more frequent and convenient flow of human capital. The exchange and interaction of systems and cultures, labor skills, and labor productivity are improved along with the diffusion of knowledge and information technology, so that residents can obtain a higher income [9, 32]. Thirdly, the opening of HSR improves the geographical location conditions of cities, reduces the spatial and temporal distance and cooperation costs, and promotes industrial cooperation between cities and the specialized division of labor based on comparative advantages. It enables small and medium-sized cities and big cities to form an industrial cooperation system with complementary advantages, promote the coordinated development of regional economy and industry, and improve residents’ income [28]. And finally, good location conditions can promote the space adjustment of the allocation of economic resources. Production factors, enterprises, and employment required by various industries tend to gather in cities along the HSR with comparative advantages, generating external economies of scale and scope, and promoting the improvement of residents’ income levels in the gathered cities [33, 34].

However, the impact of increased regional accessibility on residents’ income is uncertain. It is possible that central cities may utilize the diffusion effect to promote income growth in low-income areas [27, 35]. There may also be a "tunnel effect" in which economic activity flows from underdeveloped to developed areas and income growth is suppressed in peripheral urban areas [28].

Through a comprehensive analysis of prior scholarly works, the enhancement of regional accessibility through the implementation of High-Speed Rail (HSR) has been widely acknowledged, but there are differences in analysis scale, case regions, calculation methods and index selection. Most studies are conducted on provincial capitals or key developed cities, and comparative studies are conducted on multiple time sections, or parameter simulation and prediction are carried out. There is a lack of dynamic accessibility analysis based on the variation of actual travel time between cities on a long time series nationwide scale. Secondly, earlier studies on the impact of transport infrastructure on residents’ income were often limited to a narrow range of urban and rural areas and did not analyze the relationship between transport infrastructure and urban residents’ income at the national or regional level. For example, to explore the macro-income of residents within cities, within rural areas, or between urban and rural areas, or micro-income differences due to the heterogeneity of various influencing factors. In addition, there are differences in the direction and effect of transportation infrastructure on residents’ income, and there is still a great controversy over whether the opening of HSR and the acceleration of ordinary railway speed promote spatial polarization or equalization of regional income distribution. Thirdly, the change in regional accessibility is a set of continuous variables; the HSR is simplified as binary variables (0–1 virtual variable) in the Difference-in-Difference model, which can better evaluate the policy net effect; it ignores the improvement of accessibility in non-HSR urban areas by increasing the speed of ordinary railways; and moreover, the average impact of accessibility on income level in long time series cannot be comprehensively evaluated.

The present study employs the weighted average travel time model to calculate the dynamic changes in the minimum travel time caused by the increase in ordinary railway speed and the opening of HSR in 286 cities at prefecture level and above from 2000 to 2018. The actual variation characteristics of the accessibility of prefecture-level cities under the HSR network were accurately described by the accessibility coefficient, and the spatial distribution of the accessibility changes was analyzed by the natural discontinuity classification method of ArcGIS. Furthermore, stata.14 is used to estimate the impact of regional accessibility on urban residents’ income and its internal influencing mechanisms by using a panel model and a recursive model. It provides experience and policy inspiration for similar countries in the world on the economic effect of transportation infrastructure construction, represented by the opening of HSR and the improvement of residents’ income levels.

## Method and data

### Weighted average travel time model

The weighted average travel time model is a comprehensive index method to calculate the travel time between traffic nodes. It can reflect the accessibility level of a region through the length of travel time and the variation range in regional accessibility under the change in travel time before and after traffic development. To be specific, this index focuses on measuring the accessibility level between regions from the perspective of spatial distance, time savings or cost savings, which are affected by factors such as the location, economic strength, and density of transportation facilities of the evaluated regions. Regional accessibility is not only related to the level of spatial location and transportation infrastructure but also to the level of regional economic development and city size. The level of social and economic development affects the motivation and spatial direction of labor mobility, including travel costs and location attraction [8].

Considering the differences in railway development and economic level among 286 prefecture-level and above urban areas in China, as well as the influence of node scale and economic development level on accessibility, this paper constructs a calculation model of regional accessibility including regional minimum travel time and regional economic growth level, as shown in Eq (1):

$${\text{A}}_{\text{i}}=\frac{\sum_{\text{j}=1}^{\text{n}}\left({\text{T}}_{\text{ij}}\cdot {\text{M}}_{\text{j}}\right)}{\sum_{\text{j}=1}^{\text{n}}{\text{M}}_{\text{j}}}$$ (1)

In Eq (1), A_i_ represents the accessibility average weighted travel time of the region *i*; *i、j* represent each region, *i*,*j* = 1,2,…,286, where *i* = *j* represents the same region, and *i* ≠ *j* represents a different region; n is the total number of nodes except point i.

Eq (1) contains two elements: ① T_ij_, represents the resistance parameter between region *i* and *j*, measures the time cost of inter-regional travel, it is the shortest railway travel time between two cities, $T_{ij}=min\;\sum_{k}\;t_{ij}^{k}$. The following principles should be followed: The change of minimum travel time in urban areas with HSR is mainly caused by the opening of HSR, while the change of minimum travel time in cities without HSR is mainly caused by the speed increase of ordinary railways. If there is a direct passenger train between every two cities, the shortest rail distance of all trains is chosen; If there is no direct train between the two cities, the city with the shortest transit distance from the departure station is selected as the transit point in accordance with the principle of shortest path, and the shortest railway distance between the two cities is calculated. The shortest railway distance between the two places is divided by the fastest train speed between the two places in the corresponding year, and the speed can be dynamically adjusted according to the general speed increase of the city and the actual situation of HSR opening in the corresponding year. ② M_j_ is the comprehensive parameter of region *j*, it is used as the weighted average weight of the shortest travel time. $M_{j}=\sqrt{peo_{j}*gdp_{j}}$, which measures the attractiveness of region *j* and reflects the difference of attractiveness of other regions due to different levels of regional economy and railway development, and *peo*_*j*_ is the population size of city j, and *gdp*_*j*_ is the total GDP of city j [10, 19]. The shorter the average weighted travel time of an area, the more accessible it is.

### Coefficient of accessibility

In order to reflect the advantages and disadvantages of regional accessibility more intuitively, the accessibility coefficients were calculated based on the weighted average travel time [3], as shown in Eq (2):

$${\text{A}}^{*}{}_{\text{i}}=\frac{\sum_{i=1}^{n}A_{i}/n}{A_{i}}$$ (2)

In Eq (2), A^*^_i_ represents the accessibility coefficient of regioni, that is, the ratio between the value of urban accessibility and the average value of urban accessibility, so that the larger the accessibility coefficient A^*^_i_ is, the better the accessibility of region i is.

### Bi-directional fixed-effect panel model

Based on the improvement of regional accessibility caused by the opening and operation of HSR and the acceleration of ordinary railway, this paper uses panel model to investigate the impact of the improvement of regional accessibility on the income of urban residents in 286 cities at prefecture-level and above in China from 2000 to 2018, as shown in Eq (3):

$$\;lnc{\;}_{i,t}=\alpha_{0}+\alpha_{1}*{\text{A}}^{*}{}_{i,t}+\alpha_{2}Ind_{i,t}+\alpha_{3}Con_{i,t}+\alpha_{4}\;Ope{\;}_{i.t}+\alpha_{5}Gov_{i,t}+\alpha_{6}Edu_{i,t}+\delta_{i}+\mu_{t}+\varepsilon_{it}$$ (3)

In Eq (3), *Inc*_*i*,*t*_ is the per capita disposable income of urban residents, *i* = 1,2,⋯,286; *t* = 2000,2001,⋯,2018. A^*^_i,*t*_ is the core explanatory variable of this paper, that is, the regional accessibility index caused by the opening of HSR and the acceleration of ordinary railway. Control variables including: ①*Ind*_*i*,*t*_ represents the economic structure measured by the proportion of the output value of the secondary industry to GDP; ②*Con*_*i*,*t*_ represents the consumption scale measured by the proportion of retail sales of consumer goods in GDP; ③*Ope*_*i*,*t*_ represents openness measured by the proportion of actual foreign investment in GDP; ④*Gov*_*i*,*t*_ represents the degree of government intervention measured by the proportion of general government fiscal expenditure to GDP; ⑤*Edu*_*i*,*t*_ represents the level of human capital measured by the number of college students per 10,000 people. δ_*i*_ is a regional fixed effect; *μ*_*t*_ is time fixed effect; *ε*_*it*_ is the unobservable term.

### Data and processing

The data used in this paper are as follows: ① The economic and social panel data of 286 prefecture-level and above cities in China from 2000 to 2018 were obtained from The Statistical Yearbook of Chinese Cities from 2001 to 2019. ② The data of the shortest railway distance between regions were collected from the website of "Train Ticket" and "12306" in November 2018. The data of whether or not HSR has been opened and the speed change of ordinary railway in various cities in past years can be obtained from the website of Ministry of Railways and 12306. The data of gross regional product and year-end population are obtained from The Statistical Yearbook of Chinese Cities in 2001 and 2019. ③ The sample size of the basic data set in this paper is 5434.

The sample descriptive statistics show that the economic, demographic and social indicators of HSR cities are higher than those of cities without HSR (see Table 2). The average income of residents in cities with HSR is 18,200 yuan, while that in cities without HSR is 15,300 yuan. The average regional accessibility of cities with HSR was 1.24, while that of cities without HSR was 0.89. High-speed lines in China and site location are not random; in full consideration of development cost and economic benefit under the premise of geography, cities with a better geographical location, a higher level of economic development, a larger population, and more input from local governments are more likely to be planned for HSR lines and stations.

**Table 2:** Summary statistics, China, 2000–2018.

**Cities without HSR**		N	mean	max	min	Sd
Income	Ten thousand yuan	1558	1.53	5.67	0.28	0.89
WATT	Hours	1558	0.21	0.67	0.05	0.1
Regional accessibility	—	1558	0.89	1.87	0.26	0.31
Industry	%	1514	0.46	0.91	0.09	0.13
Consumption	%	1509	0.38	5.67	0	0.34
Open	%	1338	0.02	1.29	0	0.08
Government	%	1512	0.23	6.04	0.01	0.31
Education	people / ten thousand people	1511	0.14	1.31	0.02	0.07
**Cities with HSR**		N	mean	max	min	sd
Income	Ten thousand yuan	3876	1.82	6.8	0.33	1.1
WATT	Hours	3876	0.16	0.6	0.04	0.09
Regional accessibility	—	3876	1.24	2.63	0.39	0.37
Industry	%	3850	0.48	0.9	0.05	0.1
Consumption	%	3846	0.4	4.85	0.03	0.24
Open	%	3761	0.03	7.48	0	0.16
Government	%	3850	0.15	2.85	0	0.15
Education	people / ten thousand people	3845	0.14	1.86	0	0.06

## Results

### Regional accessibility

**Fig 3:**
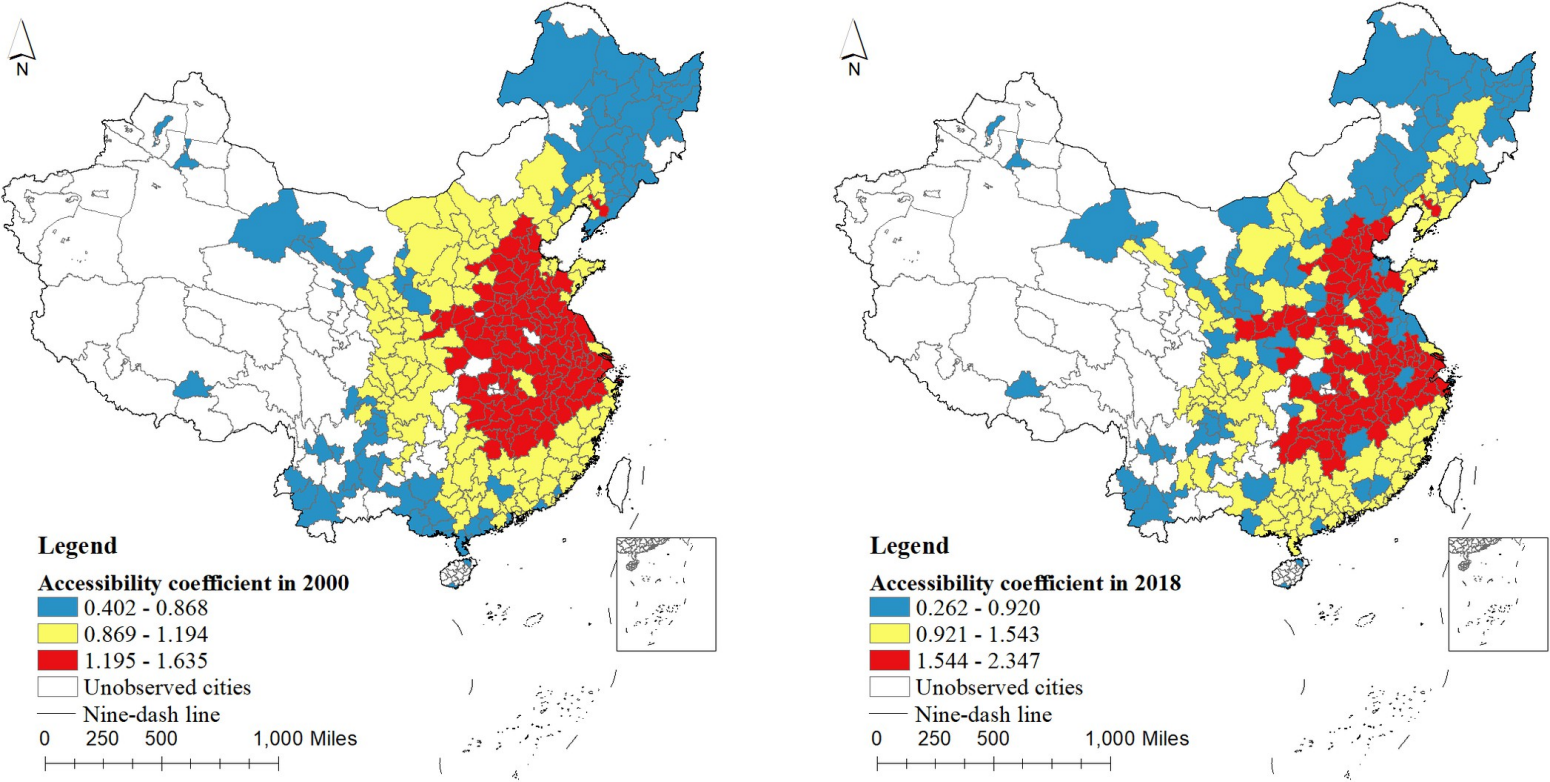
Regional accessibility coefficients of 286 cities at prefecture level and above in China in 2000 and 2018. Note: ① The base drawing is derived from the open maps of China’s national natural resources standard map service website (http://bzdt.ch.mnr.gov.cn/), the drawing number is GS (2016) 2923. This map does not change the content of the base drawing and is only used as a schematic diagram.② Cities are divided into three categories according to Jenks grading method of natural discontinuities in ArcMap10.2. Regional accessibility is measured by accessibility coefficient, and the greater the value, the better the regional accessibility.

Fig 3 shows that according to the regional accessibility coefficient, 286 cities at prefecture-level and above in China are divided into three categories of "high, medium and low". In 2000, the regional accessibility coefficients of 286 cities in China showed a trend of concentrated distribution, with the highest in the eastern region, followed by the central region, and the lowest in the western and northeast region. In 2018, the spatial distribution polarization trend of regional accessibility coefficients in 286 cities weakened, that is, the accessibility of urban regions generally improved.

In the year 2018, there was a noticeable reduction in the spatial distribution polarization trend of regional accessibility coefficients across 286 cities. This indicates a trend towards greater uniformity in accessibility, resulting in an overall improvement in the accessibility of urban regions.

### Estimation of the impact of regional accessibility on income

In Table 3, mixed regression (Pooled regression) was first used as the reference frame for estimation. There may be missing variables that do not change over time because each city is different, fixed effects model regression (FE) is needed. All individual dummy variables of fixed effects regression (LSDV) are significant (P value is 0), so the hypothesis that all individual dummy variables are 0 is rejected. There is an individual effect, and mixed regression is not applied, that is, a fixed effect is better than a mixed effect. The time effect was considered in the fixed effect model, and two-way fixed effect model regression (two-way FE) was carried out. However, individual effects may exist in the form of random effects (RE). The LM test for individual-specific effects strongly rejects the null hypothesis of "no individual random effects" (P value is 0), that is, random effects are better than mixed effects. The results of the over -identification test showed that the χ^2^(6) statistic was 221.353 and the P value was 0.000This outcome provides strong evidence for the rejection of the random effect, thereby suggesting that the fixed effect is more dominant than the random effect. In summary, the fixed effects regression results (see Table 3 (4)) are optimal. The increase of 1 unit in regional accessibility coefficient brought by the opening of HSR and the acceleration of ordinary railways has increased the per capita disposable income of urban residents by 2140 yuan on average.

**Table 3:** Impact of regional accessibility improvement on urban residents’ income, China, 2000–2018.

	Panel estimation				Robustness test(PSM)		Instrumental variable test	
	(1)	(2)	(3)	(4)	(5)	(6)		(7)
	Pooled	RE	FE-LSDV	Two-way FE	Kernel	K(4)		IV
**Regional accessibility**	0.670***	1.396***	1.998***	0.214***	0.211***	0.185***	**Second stage regression**	
	(0.088)	(0.120)	(0.173)	(0.059)	(0.058)	(0.059)		
**Industry**	0.010***	0.001	-0.005	-0.012***	-0.012***	-0.010***	**dum_HSR**	0.706***
	(0.003)	(0.004)	(0.005)	(0.002)	(0.002)	(0.002)		(0.221)
**Consumption**	-0.003	-0.546***	-0.841***	-0.026	-0.012	-0.044	**N**	4963
	(0.157)	(0.181)	(0.211)	(0.057)	(0.057)	(0.057)	***R*** ^***2***^	0.937
**Open**	0.029	-0.147	-0.191	-0.016	-0.050	-0.012		
	(0.108)	(0.163)	(0.180)	(0.025)	(0.045)	(0.035)	**First stage regression**	
**Government**	1.876***	2.919***	3.392***	-0.444***	-0.475***	-0.416***		
	(0.255)	(0.356)	(0.442)	(0.097)	(0.102)	(0.089)	**Instrumental variable**	0.255*
**Education**	-2.680**	-3.823***	-4.094***	0.446***	0.440***	0.324**		(0.045)
	(1.250)	(1.173)	(1.276)	(0.144)	(0.143)	(0.130)	**RKF statistic**	31.878
**_cons**	0.587**	0.368	0.508	0.839***	0.837***	0.757***		
	(0.241)	(0.275)	(0.335)	(0.107)	(0.107)	(0.104)	**F statistic**	455.021
**Time-fixed**	No	No	No	Yes	Yes	Yes		
**Regional-fixed**	Yes	Yes	Yes	Yes	Yes	Yes	**Control variables**	Yes
**N**	5076	5076	5076	5076	5074	3675	**Time-fixed**	Yes
**R** ^**2**^	0.184	0.523	0.513	0.944	0.944	0.945	**Regional-fixed**	Yes

Note: ① In parentheses are clustering robust standard errors for cities.②*, **, *** represent significant at the significance level of 10%, 5% and 1% respectively.

### Propensity Score Matching (PSM) method test

The selection of HSR routes and stations is not random. In order to avoid sample selection bias, the Propensity Score Matching (PSM) method is further applied to control the self-selection effect of a city with or without HSR. 6 Matching indicators [36] consistent with the baseline regression control variables were applied to obtain the probability value of the operation of HSR in a city through the Kernel Matching method of the Probit model (see Table 3 (5)) and the k-nearest neighbor Matching method (k(4), see Table 3 (6)). Cities already operating HSR are matched with similar non-HSR cities. The robustness test results prove the robustness of the benchmark regression in this paper.

### Instrumental variable method test

The endogenous problems caused by local preferences such as good economic foundation or significant regional advantages in HSR construction can be identified by the method of instrumental variables. The tractive force of railway will decrease significantly with the increase of surface steepness in the region through which it passes, that is, the railway construction cost will increase significantly. A strictly exogenous variable of Least Cost Path Spanning Tree Networks is built as an instrument variable for HSR opening [37] through elevation data extraction, with municipalities, provincial capitals and sub-provincial cities as target cities. The route network based on the principle of the lowest geographical development cost is obtained, and the dummy variable (0–1) of "whether there should be HSR service" in each city is calculated, as shown in Eq (4):

$$\;Cost_{i}=0.3\;water_{i}+0.4slope_{i}+0.3\;grads_{i}$$ (4)

In Eq (4), *Cost*_*i*_ is the instrumental variable, representing the lowest geographic development cost. *water*_*i*_ represents urban hydrological information. *slope*_*i*_ represents urban slope information; *grads*_*i*_ represents urban fluctuation.

The regression results of the first stage of the two-stage least square method (2SLS) show that the instrumental variables have a significant positive impact on the income. F value is greater than 10, Kleibergen-Paap rk Wald F statistic value is greater than 10, the hypothesis of weak tool variable is rejected, and the validity of instrumental variable is proved, which also verifies the robustness of the research results in this paper(see Table 3 (7)).

## Discussion

### Income effect of rail

The baseline regression results show that the improvement of regional accessibility caused by the opening of HSR and the acceleration of ordinary railways can promote urban residents’ income growth. The geospatial organization of economic activities mainly depends on the transportation infrastructure system, which is an important channel for the spatial flow of economic elements and the basis for effective connection and strengthening of regional connections [38]. Traffic conditions affect the flow of labor between departments, regions and configuration. The operation of HSR can greatly improve the accessibility of the area city; it will reduce transportation cost directly, promote the flow of inter-city physical and human capital, and alleviate the misconfiguration of resources. According to the theory of labor supply and demand, HSR brings convenience to residents’ employment and lives, and will increase the attractiveness of the region and labor supply. On the other hand, it will bring productivity growth to enterprises and increase their demand for labor. If the increased infrastructure has a large productivity growth effect on enterprises, the increase in enterprise demand for labor will be greater than the increase in labor supply, leading to a rise in regional wages [39].

The influence coefficient of economic structure is significantly negative, indicating that the secondary industry has limited effect on the improvement of residents’ income. The social and economic structure needs to develop towards the tertiary industry to achieve the rationalization and upgrading of the tertiary industrial structure, which is more conducive to the improvement of urban residents’ income level. HSR can promote the polarized development of the modern service industry in big cities and the balanced development of secondary industry between small and medium-sized cities and big cities. It can help some regions transform from traditional industries to service industries focusing on tourism and business [40]. To form an agglomeration of producer services mainly with highly skilled labor and life services mainly with low-skilled labor to promote the income growth of urban residents [41]. The influence coefficient of government size is significantly negative, indicating that the smaller the proportion of government expenditure, the higher the openness of market economy and the more conducive it is to improving the income levels of urban residents. The influence coefficient of human capital level is significantly positive, and human capital is an important factor affecting residents’ income level [42].

### Regional heterogeneity

There is regional heterogeneity in the moderating effect of regional accessibility on the income of urban residents in different regions of China (see Table 4(8)-(11)). China’s eastern coastal cities (such as the Pearl River Delta and Yangtze River Delta) are in an advantageous position in terms of geographical location, trade level and enterprise productivity, and have achieved rapid transformation and upgrading of their economic and industrial structures. The transformation and upgrading of the economic and industrial structure were achieved fairly quickly, and advanced manufacturing, high-end service industries, and strategic emerging industries were concentrated. Among them, the value-added of the tertiary industry contributed far more to economic growth than the primary and secondary industries. Good regional economic development raises the corresponding income of residents to a high level, and these regions become the regions with the highest income of urban residents in China. In addition to wage income, housing prices in the first and second-tier eastern cities have continued to rise in recent years, driving the increase of residents’ net property income and transfer net income directly. The favorable environment for innovation and entrepreneurship also promotes the increase of residents’ business income.

**Table 4:** Regional heterogeneity of the impact of regional accessibility on urban residents’ income, China, 2000–2018.

	Eastern China	Central China	Western China	Northeastern China	Central cities	Peripheral cities
	(8)	(9)	(10)	(11)	(12)	(13)
Regional accessibility	0.320**	0.084	0.100	0.463***	0.465**	0.130**
	(0.122)	(0.058)	(0.075)	(0.137)	(0.224)	(0.056)
Industry	-0.034***	-0.002	-0.008***	-0.000	-0.031***	-0.009***
	(0.004)	(0.002)	(0.003)	(0.002)	(0.011)	(0.002)
Consumption	-0.256	-0.077	-0.002	0.188**	-0.077	0.018
	(0.184)	(0.067)	(0.043)	(0.083)	(0.105)	(0.058)
Open	0.098	0.018	0.014*	-0.105	-0.051**	-0.047
	(0.097)	(0.028)	(0.007)	(0.093)	(0.021)	(0.042)
Government	-0.687*	-0.107	-0.336***	-0.672***	-0.351	-0.437***
	(0.361)	(0.132)	(0.099)	(0.218)	(0.378)	(0.101)
Education	1.268**	0.124	0.011	2.671*	1.550	0.383***
	(0.530)	(0.156)	(0.119)	(1.321)	(1.333)	(0.132)
_cons	1.851***	0.486***	0.756***	-0.275	1.389**	0.770***
	(0.275)	(0.130)	(0.142)	(0.240)	(0.632)	(0.100)
Time-fixed	Yes	Yes	Yes	Yes	Yes	Yes
Regional-fixed	Yes	Yes	Yes	Yes	Yes	Yes
*N*	1644	1505	1295	632	545	4531
*R* ^2^	0.941	0.961	0.974	0.967	0.955	0.948
*p*					0.001***	

Note: ① In parentheses are clustering robust standard errors for cities. ②*, **, *** represent significant at the significance level of 10%, 5% and 1% respectively. ③ Empirical P-values showed the significance of coefficient differences between groups, which were estimated by Bootstrap 1000 times using Fisher’s Permutation test. ④ Central cities refer to municipalities directly under the central government, provincial capitals and vice-provincial cities, while other cities are peripheral cities.

Recently, the central and western regions have actively pursued the industrial transfer from the eastern region, which has, to a certain extent, realized the transformation of the development mode of new economy and new industries and the improvement of core competitiveness and economic growth quality, and promoted the growth of residents’ income. However, there is still a certain gap between the income level of residents in the developed cities in the east [43]. Northeast China is the region with the earliest start of industrialization in China. Northeast China has become a region with high economic development, urbanization level and residents’ income level in China, relying on a solid industrial foundation and rich resources. In recent years, its economic growth has gradually declined, and residents’ income has grown slowly due to unfavorable factors such as a rigid industrial structure and economic system. However, the improvement of location conditions brought by the development of transportation infrastructure has created new development opportunities, such as economic growth and income increase brought by abundant environmental resources and snow and ice tourism [19, 44].

The coefficient of regional accessibility has a positive effect on the income of residents in both central and peripheral cities (See Table 4 (12) ‐ (13)). According to the empirical P value, the difference of the influence coefficient between the central city and the peripheral city is statistically significantly different from zero, which can be compared between groups. That is, the improvement of the accessibility coefficient of the central city has a higher effect on the income of urban residents than that of the peripheral city. The non-equilibrium of the HSR network results in uneven benefits of urban transportation network construction and changes in urban relative location [45–47]. Some central cities, as the gathering points of HSR, often have a strong economic foundation, and the development of HSR will further strengthen the economic potential of urban agglomerations or advantageous regions [48]. The opening and operation of HSR have strengthened economic ties between regions, promoted resource and service sharing among cities, accelerated regional economic integration, and promoted the development of urban agglomerations from single to multi-centers. The external radiation capacity of central cities has been constantly enhanced. It can bring all the regional production sectors in the whole economic system into a large division of labor structure system of production and trade driven by nearby towns and narrow the economic development gap between regions and the income gap of residents through the diffusion effect [34].

### Mechanism analysis

A recursive model [49] is further constructed to identify the conduction path of the mediating effect of regional accessibility improvement on urban residents’ income, as shown in Eqs (5) ‐ (7):

$$Inc_{i,t}=\rho_{0}+\rho_{1}*{\text{A}}^{*}{}_{i,t}+\gamma x_{i,t}+\delta_{i}+\mu_{t}+\varepsilon_{i,t}$$ (5)

$$Med_{i,t}=\beta_{0}+\beta_{1}*{\text{A}}^{*}{}_{i,t}+\gamma x_{i,t}+\delta_{i}+\mu_{t}+\varepsilon_{i,t}$$ (6)

$$Inc_{i,t}=\delta_{0}+\delta_{1}*{\text{A}}^{*}{}_{i,t}+\delta_{2}*Med_{i,t}+\gamma x_{i,t}+\delta_{i}+\mu_{t}+\varepsilon_{i,t}$$ (7)

In Eqs (5) ‐ (7), *Med*_*i*,*t*_ indicates the intermediary variable, which is the number of employment and the total fixed asset investment respectively. *x*_*i*,*t*_ is a group of covariables identical with the baseline regression [36].

The results show that there is a mediation effect between the change of regional accessibility and the income of urban residents. Under the adjustment of employment, regional accessibility has a significant impact on resident income (see Table 5 (14) ‐ (16)), and the proportion of the mediating effect to the total effect is 0.223. Regional accessibility has a significant impact on residents’ income adjusted by fixed asset investment (see Table 5 (14), (17) ‐ (18)), and the proportion of the mediating effect to the total effect is 0.242. Labor employment is a mediating variable of regional accessibility affecting income. Labor tends to migrate to regions with more economic opportunities and higher wages until inter-regional economic opportunities tend to be balanced [50–52]. The urban income level will be significantly increased when employment reaches a certain level. Fixed asset investment can increase market vitality, expand employment, and promote sustainable economic development effectively. However, the development of fixed asset investment is unbalanced among regions, and the range of investment and the yield rates are also significantly different. The performance of regional accessibility can directly improve the income of residents in the region, and at the same time, regional income can be improved by increasing regional fixed asset investment [28].

**Table 5:** The mediating effect of regional accessibility on residents’ income, China, 2000–2018.

	Income	Mediating effect of employment		Mediating effect of fixed asset investment	
		Employment	Income	Total investment in fixed assets	Income
	(14)	(15)	(16)	(17)	(18)
Regional accessibility	0.214***	29.214***	0.166***	1259.076***	0.162***
	(0.059)	(5.915)	(0.055)	(235.420)	(0.060)
Employment			0.002***		
			(0.001)		
Total investment in fixed assets					0.000
					(0.000)
Industry	-0.012***	-0.666***	-0.011***	-19.544**	-0.012***
	(0.002)	(0.191)	(0.002)	(8.148)	(0.002)
Consumption	-0.026	-6.748	-0.015	271.091	-0.038
	(0.057)	(5.314)	(0.052)	(201.401)	(0.053)
Open	-0.016	1.301	-0.018	117.169**	-0.021
	(0.025)	(2.108)	(0.023)	(59.272)	(0.025)
Government	-0.444***	-10.197	-0.428***	-1.3e+03***	-0.390***
	(0.097)	(7.729)	(0.093)	(364.959)	(0.094)
Education	0.446***	25.673	0.404***	8.166	0.446***
	(0.144)	(18.305)	(0.131)	(402.181)	(0.140)
_cons	0.839***	44.323***	0.767***	-526.067*	0.861***
	(0.107)	(11.033)	(0.109)	(304.907)	(0.104)
Mediation mechanism			Partial mediation		Partial mediation
The proportion of mediating effect to total effect			0.223		0.242
Time-fixed	Yes	Yes	Yes	Yes	Yes
Regional-fixed	Yes	Yes	Yes	Yes	Yes
N	5076	5076	5076	5075	5075
R^2^	0.944	0.150	0.946	0.358	0.946

Note: ① In parentheses are clustering robust standard errors for cities. ②*, **, *** represent significant at the significance level of 10%, 5% and 1% respectively.

## Conclusions

The unbalanced regional economic development and the continuous differentiation of income gap are the problems existing simultaneously in China’s current economic development. It is imperative to address unbalanced and inadequate development, and narrow disparities in regional development and income distribution to promote common prosperity.

This paper uses the weighted average travel time model and accessibility coefficient method to estimate the changes in accessibility in 286 cities at prefecture-level and above from 2000 to 2018, based on the opening of HSR and the speed increase of ordinary railway. Then, the influence and internal mechanism of improving regional accessibility on the income of urban residents are estimated by using the bidirectional-fixed effects panel model and the recursive model, respectively.

The results show that: (1) The accessibility of urban areas has been greatly improved due to the opening of HSR and the acceleration of ordinary railway, among which the improvement of HSR cities is greater. (2) The improvement of regional accessibility significantly promoted the income growth of urban residents. (3) There is regional heterogeneity in the impact of regional accessibility improvement on urban residents’ income, it has a significant promoting effect on the eastern and northeastern regions. It has a greater positive effect on improving the income of residents in central cities compared with peripheral cities. (4) Regional accessibility can promote urban income growth through regional employment and fixed asset investment.

China should pay more attention to the adjustment effect of large-scale transportation infrastructure investment on residents’ income between regions. Coordination is an inherent requirement for sustained and sound economic and social development. To achieve substantive progress in achieving common prosperity across the board, we must focus on promoting coordinated development among regions, urban and rural areas, all social strata, and all industries, and make development more balanced on the whole. China’s future urban development will form a network urban agglomeration structure of "multi-center, multi-level and multi-node". Central cities and urban agglomerations are becoming the main spatial forms of carrying development factors in China. The development of transportation infrastructure has broken through the central-marginal mode under the previous geographical restrictions and become an important way to narrow the income gap and improve the welfare of residents. The space-time contraction effect brought by HSR has greatly promoted the change of regional spatial patterns, helped eliminate regional barriers, promoted the flow of resource elements, and improved resource allocation and production efficiency. Further optimization of urbanization’s spatial pattern and urban scale structure will be conducive to better playing the economic agglomeration advantages of big cities and central cities, improving the functional professional level of small and medium-sized cities, promoting the evolution of the division of labor among cities, and promoting regional economic integration. This will improve the income gap between regions and promote common prosperity of the people. The effect of HSR network development on improving urban location has gradually exceeded the influence of urban location space on urban accessibility, but there are huge differences in the degree of improvement in urban accessibility in different regions of China.

In order to make full use of the development opportunity of transportation infrastructure to improve the income gap of regional residents, this paper puts forward the following experience and enlightenment: Firstly, more attention should be paid to the spatial balance of the transportation network in the layout of HSR lines. Secondly, the integrated construction of a transportation network can be realized, and the continuous degree of regional accessibility can be improved to expand the flow of production factors. Thirdly, peripheral cities should reasonably plan their own industrial development layout and realize complementary advantages and division of labor cooperation with central cities.

The main contributions of this paper are as follows: Firstly, this paper comprehensively considered the dynamic process in which 204 out of 286 cities in China have successively opened and operated HSR from 2008 to 2018 and the dynamic change in which ordinary railways have experienced four times the speed increase from 2000 to 2018. The shortest railway travel time between cities and the accessibility of each urban region were calculated by a weighted average travel time model and accessibility coefficient, so as to accurately estimate urban accessibility and its dynamic temporal characteristics over the years. Secondly, the bidirectional-fixed effects panel model can overcome the simplification of policy dummy variables of the difference-in-difference model and more fully estimate the time series and cross-section characteristics of panel data. Thirdly, this paper analyzed the mechanism of regional accessibility improvement affecting residents’ income, which can provide a reference for better identifying the focus point of policy.

This paper has the following limitations: The weighted average travel time model, considering the frequency of train departure, stop, and arrival, can more fully estimate the size of the transportation capacity of node cities. However, the train frequency data of stations is real-time data, and the historical data of previous years cannot be obtained from the current website. The macro-level data on urban per capita disposable income from 2000 to 2018 were used as a proxy variable for urban residents’ income level without the non-continuous income survey data from micro-databases such as China Family Panel Studies (CFPS). We didn’t identify the movement of labor or residents between cities and the resulting changes in individual incomes. In future research, the data on train station stop frequency will be manually combed from the railway train schedule published in previous years so as to calculate a more accurate index of regional accessibility. In addition, the income data from the micro survey will be combined to form mutual evidence at the macro and micro levels, which will further enrich this research.

## Supporting information

S1 File The data used in this study. (XLSX)
